# Comparing the effectiveness of augmented reality-based and conventional instructions during single ECMO cannulation training

**DOI:** 10.1007/s11548-021-02408-y

**Published:** 2021-05-23

**Authors:** Julian Wolf, Viviane Wolfer, Maximilian Halbe, Francesco Maisano, Quentin Lohmeyer, Mirko Meboldt

**Affiliations:** 1 Product Development Group Zurich, ETH Zurich, Leonhardstrasse 21, 8092 Zurich, Switzerland; 2 University Heart Center, University Hospital Zurich, Rämistrasse 100, 8091 Zurich, Switzerland; 3 University of Zurich, Pestalozzistrasse 3/5, 8091 Zurich, Switzerland

**Keywords:** User study, Mixed reality, User guidance, Medical training

## Abstract

**Purpose:**

Effective training of extracorporeal membrane oxygenation (ECMO) cannulation is key to fighting the persistently high mortality rate of ECMO interventions. Though augmented reality (AR) is a promising technology for improving information display, only a small percentage of AR projects have addressed training procedures. The present study investigates the potential benefits of AR-based, contextual instructions for ECMO cannulation training as compared to instructions used during conventional training at a university hospital.

**Methodology:**

An AR step-by-step guide was developed for the Microsoft HoloLens 2 that combines text, images, and videos from the conventional training program with simple 3D models. A study was conducted with 21 medical students performing two surgical procedures on a simulator. Participants were divided into two groups, with one group using the conventional instructions for the first procedure and AR instructions for the second and the other group using instructions in reverse order. Training times, a detailed error protocol, and a standardized user experience questionnaire (UEQ) were evaluated.

**Results:**

AR-based execution was associated with slightly higher training times and with significantly fewer errors for the more complex second procedure ($p<0.05$, Mann–Whitney *U*). These differences in errors were most present for knowledge-related errors, resulting in a 66% reduction in the number of errors. AR instructions also led to significantly better ratings on 5 out of the 6 scales used in the UEQ, pointing to higher perceived clarify of information, information acquisition speed, and stimulation.

**Conclusion:**

The results extend previous research on AR instructions to ECMO cannulation training, indicating its high potential to improve training outcomes as a result of better information acquisition by participants during task execution. Future work should investigate how better performance in a single training session relates to better performance in the long run.

## Introduction

Extracorporeal membrane oxygenation (ECMO) is a life-saving procedure for severe respiratory or cardiac failure that has evolved from a last-resort treatment to a more mainstream therapy over the past few years [10]. As ECMO cannulations gain more importance as an emergency treatment, the number of cases is increasing rapidly and more and more hospitals are performing ECMO cannulations themselves [7]. While the mortality rate has decreased slightly as ECMO usage has increased, it remains high at over 60% [13].

Sufficient training for ECMO cannulation in general is shown to be linked with decreasing mortality rates [14]. Furthermore, simulation-based training shows significant improvements for ECMO cannulations [2, 6]. Frequent and realistic training therefore seems to be key when it comes to successful ECMO procedures. However, training is time-consuming, and there is no standardized certification or training process for ECMO cannulations [6]. Even though the Extracorporeal Life Support Organization (ELSO) has developed specific guidelines for safe ECMO practice, they are only used as a basic structure for ECMO centers to build varying institution-specific guidelines and programs around [6, 8]. Since ECMO cannulations are often emergency operations, a physician can go months or years without having to perform an ECMO cannulation before suddenly being confronted with a time-critical situation [19]. To be able to act precisely and quickly, physicians need to be provided with frequent and thorough training.

As a consequence, several approaches have been put forward, aiming to simplify and improve ECMO cannulation training options on the one hand and to work toward a standardized procedure on the other. Simulation-based medical training in general is swiftly gaining ground, and high-fidelity simulators have been developed, with promising results, for ECMO cannulations as well [19]. Many of these involve mannequins and/or silicon-based tissue pads to make for realistic cannulation training [17]. Other simulators even involve the recreation of the ECMO circuit and include applications where the instructor can manipulate ECMO data, among other factors, to simulate common problems [1]. Those, however, are mainly designed for post-cannulation problems.

In other areas of medicine, augmented reality (AR) has started to emerge as a training tool in recent years, offering fundamentally new possibilities for visualization and interaction with digital content. Modern devices (e.g., the Microsoft HoloLens 2) are affordable and easy to use and therefore widely accessible for training purposes. However, only a small percentage of AR projects have dealt with training procedures, while most of them have been applied to actual treatment scenarios [9]. Existing training applications based on AR depict, for example, the internal anatomy superimposed on a simulator [12] or overlay a CT scan to train methods for ultrasound [5]. Currently, there is no work on how optical head-mounted displays (OST-HMD) can be utilized for ECMO cannulation. Furthermore, only limited research on step-by-step procedures and on the possible benefits of AR display options in medical training has been conducted. Azimi et al. [3] first trained and then assessed 20 participants in two emergency medical procedures to compare the effectiveness of AR-based instructions provided by an OST-HMD to conventional training. They found participants using the AR instructions spent more time training but were faster in completing the procedure in the assessment run. Participants further found the use of OST-HMDs more engaging and reported higher levels of confidence.

In this article, we evaluate AR step-by-step instructions for ECMO cannulation training and compare them with the conventional training instructions regularly used at a university hospital. In addition to training times and user experience, we emphasize the quality of execution, i.e., the number of errors, observed in a single training run. Fewer errors during the training are expected to lead to fewer necessary training iterations and less supervision needed to learn the procedure.

We see two chief advantages of AR step-by-step instructions: learners’ access to (1) contextual information, which reduces complexity to manageable increments, and (2) the proximity of information, which allows participants to continuously check their execution against the tutorial in real time and to adjust their behavior accordingly. Consequently, we expect AR training instructions to result in shorter training times, fewer errors, and better user experience and thus lead to higher skill levels after a single training run.

## Related works

Even though AR applications for ECMO cannulation training have not yet been evaluated, previous work has investigated the benefits of ECMO cannulation training as well as AR for medical training.

### Augmented reality in medical training

AR is used and has been evaluated in different areas of medicine, including surgical environments [22], therapy [11], and training [4]. In [18], medical training with mobile AR was compared to textbook-based learning. Medical students were asked to study the given material (either mobile AR or textbook) for 45 minutes. No significance differences between the two groups were reported for either knowledge tests or experience questionnaires, but indicated that long-term retention of knowledge may be better with mobile AR. In contrast to the present study, the study only tested medical knowledge and no manual execution of steps.

Other work with Microsoft HoloLens 1 investigated the differences between AR-based and computer-based suture training for medical students [16]. Participants could choose to watch videos on either the HoloLens or a conventional computer and to execute a suturing pattern. The study showed that videos were watched more frequently on the HoloLens, but there was no significant difference regarding the execution of or time spent on the manual task.

### ECMO cannulation training

Several studies have shown that adequate cannulation training is crucial for minimizing complications and is therefore an important topic for further investigation and improvement [14, 20]. High-fidelity cannulation simulators have been developed, and various studies indicate that simulation-based training for ECMO implantations is highly effective for participants’ knowledge and ability and hence results in lower cannulation time and better execution [2, 6].

## Methods

### Study design

To compare conventional and AR-based instructions for ECMO cannulation training, a study was conducted with 21 medical students. Participants had to perform two procedures, each using a different mode of instruction. Similar to the study performed by Azimi et al. [3], participants were split into two groups. One group performed P1 (procedure 1) with AR instructions and P2 with conventional instructions, while the other group performed the same training, but in reverse. Hence, each group was acting as the control group for one procedure. To minimize sequence effects of procedures, half of the participants of each group started with the second and the other half with the first procedure.

**Fig. 1:**
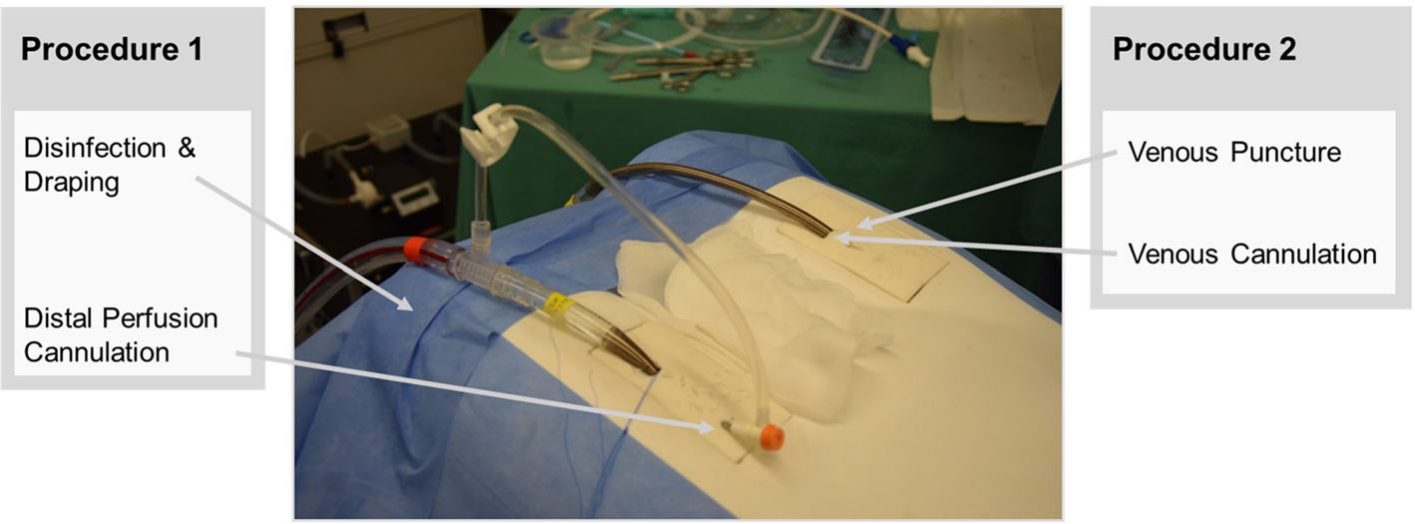
The two procedures investigated during the study. Each procedure is performed on a different side of the simulator

**Fig. 2:**
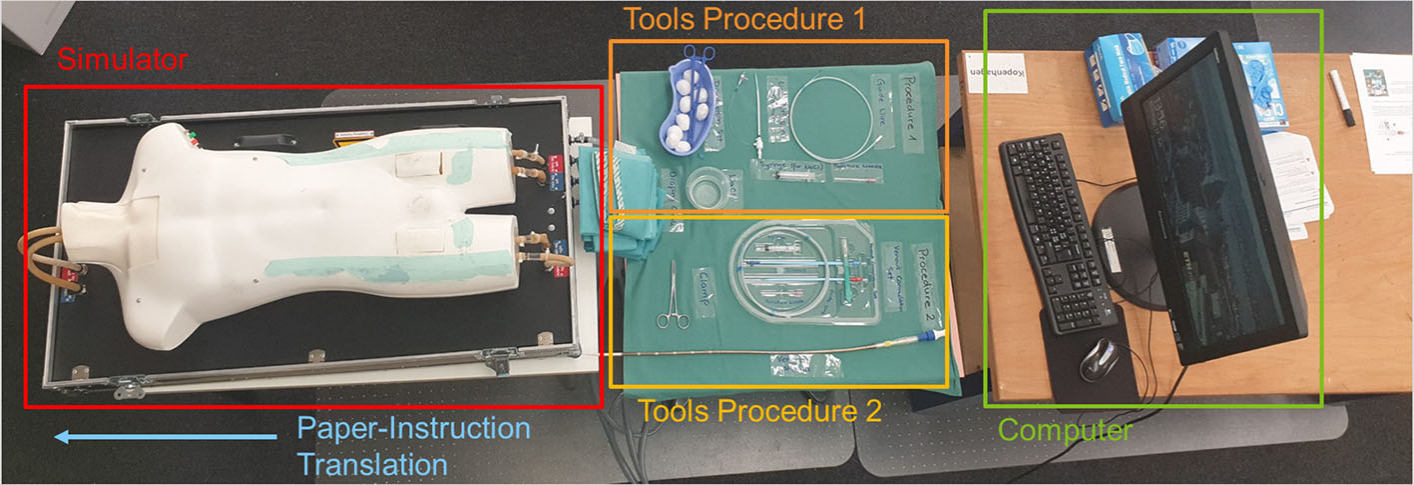
Experimental setup consisting of a simulator, tools for procedure 1 and 2, and a desktop computer. Paper instructions are placed on the left-hand side of the simulator

**Fig. 3:**
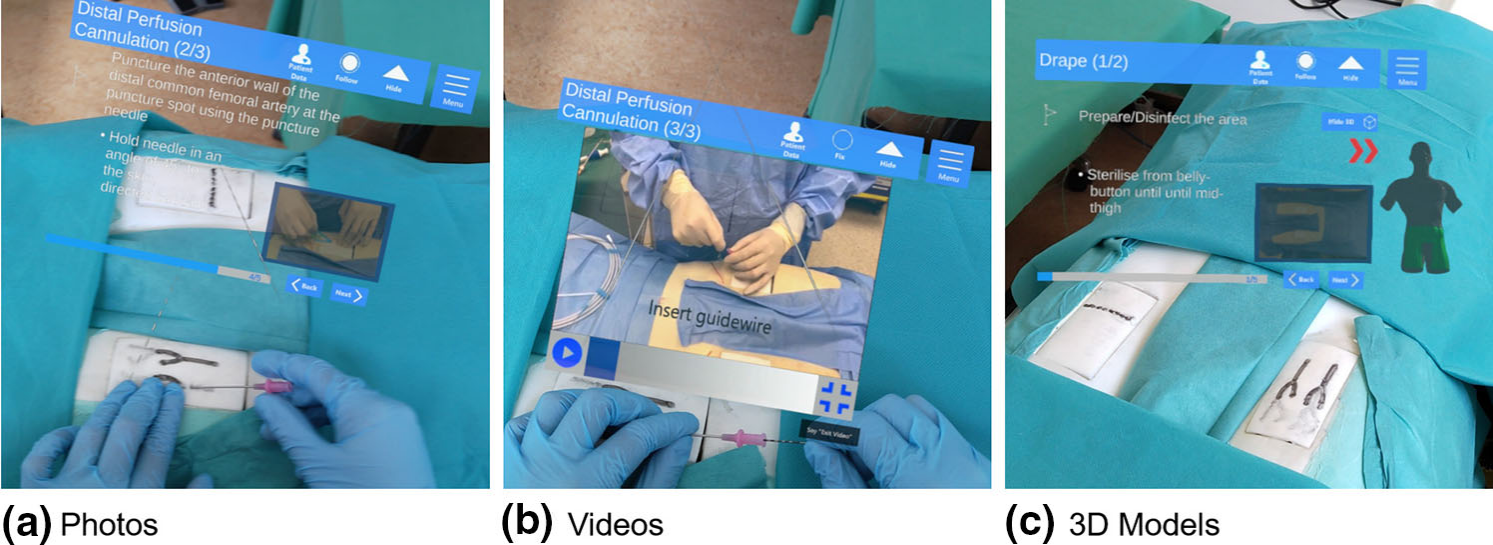
Three information representation types complementing the text-based step-by-step instructions in AR

#### Task

Two surgical procedures were performed by each participant (Fig. 1). The procedures were adapted to the study framework so that all steps could be completed on the simulator. Steps including ultrasound verification alone were skipped, and steps including ultrasound guidance were adapted accordingly. More precisely, prior to the experiment, we drew the bifurcations (venous and arterial) for the distal perfusion cannulation and marked the height of the puncture spot for the venous cannulation.

Distal perfusion cannulation included the identification of the left femoral artery branching, the puncture of the left distal common femoral artery (directed caudally), insertion of the limb perfusion cannula using the “Seldinger Technique”, removal of the guidewire, and an NaCl flush. Venous puncture comprised the identification of the right femoral vein, the incision of the skin at the puncture spot, puncture of the common femoral vein (directed cranially), and the measurement of the required cannula and wire length. Finally, venous cannulation included advancing the wire to the measured length, serial dilation of the vessel (using 3 dilators), the insertion of the venous cannula, and the removal of the wire and clamping of the cannula.

#### Experimental procedure

Participants were first asked to fill out a questionnaire regarding their previous experiences with HoloLens and ECMO devices as well as a consent form. They then went through a ten minute HoloLens 2 tutorial to familiarize themselves with the AR interface and the different navigation types (voice command and hand gesture). Participants then performed both procedures. Prior to starting a procedure, participants watched a video showing all steps to be performed, which aimed at imitating a live-demonstration in real surgery prior to training. After each procedure, they filled out a user experience questionnaire (UEQ).

#### Experimental setup

The setup consisted of a simulator, a table with a green table cloth on which all tools were placed, and a stationary desktop computer (Fig. 2). The simulator was placed in such a way that the hoses connected to it did not disturb the participant. All tools were labeled with the tool names used in the paper or AR instructions. They were divided into two sets, one for procedure 1 and the other one for procedure 2. A desktop computer was placed next to the tools, on which participants filled out the questionnaires and watched the initial videos. During the conventional training, they could also use the computer to watch the video sequences presented in the AR instructions.

#### Participants

The study was conducted with 21 third- and fourth-year medical students (aged 22–30, 9 males, 12 females) who had never performed an ECMO cannulation before.

### Information material

Both conventional and AR instructions are based on a standard operating procedure for ECMO cannulation developed at the heart center of a university hospital. Some adjustments were made to the instructions to better suit the simulation setting. This included the replacement of ultrasound guidance with predefined marks on the simulator and some additional specifications to make the cannulation possible for participants with no previous ECMO experience.

#### Conventional instructions

Conventional instructions consist of a printed version of the standard operating procedure that include text and supplementary images. In addition, participants could watch video sequences on a desktop computer (cf. Fig. 2).

#### Augmented reality instructions

The AR instructions were designed as a step-by-step guide that included the same text, pictures, and videos as the conventional instructions. In addition, simple 3D models were displayed in two steps (Fig. 3). It was developed for the Microsoft HoloLens 2 (Microsoft, Redmond, Washington) using the Unity 3D Game Engine (Unity Technologies, San Francisco, California). The application can be controlled both by hand gestures and voice commands. Using the outstretched index finger, the user can interact with the interface simply by moving the finger “through” the projected button in the same way one would press a physical button. Audio as well as visual feedback indicates that a button was successfully pressed. Voice commands work either by reading out the name of a particular button or by focusing the eyes on the button and saying “select”.

### Simulator

A TF200 ECMO-Simulator by Erler Zimmer (AcuMax Med AG, Bad Zurzach, Switzerland) was used. It is specifically designed for ECMO cannulation training and regularly used during trainings at the university hospital. The simulator contains venous and arterial blood circulation through a hose system. Integrated pumps allow for individually adjustable blood flow and therefore realistic simulation. Common medical tools can be used on the simulator.

### Data analysis

For the comparison of conventional and AR-based instructions, training time, number of errors, and user experience were evaluated.

#### Error analysis

We derived an error protocol that contains a list of possible errors and ranks each of them with a factor between 1 and 3 based according to their severity.

- **1-Point-Errors**

- Small errors
- Errors without impact on further steps
- Partially completed sub-steps

- **2-Point-Errors**

- Larger errors
- Errors with impact on other steps or further progression

- **3-Point-Errors**

- Skipped steps
- Maximum time for a step exceeded (leading to incompletion)

Errors were further categorized as either handling errors, knowledge errors, or both. Handling errors are more related to participants’ individual dexterity than to the clarity of the instructions, while knowledge errors are caused by the participants’ lack of attention to the relevant, task-related information. The error protocol for procedure 2, including all errors and their respective categorization and severity ranking, is shown in Fig. 6.

#### User experience questionnaire

To assess the participant’s personal experience, a User Experience Questionnaire (UEQ) [15] was handed out after each procedure. This standardized questionnaire consists of 26 questions. For each question, two contrasting adjectives were juxtaposed and the participant was asked to decide where on the scale, from 1 (complete agreement with the left adjective) to 7 (complete agreement with the right adjective) their personal experience lay.

**Fig. 4:**
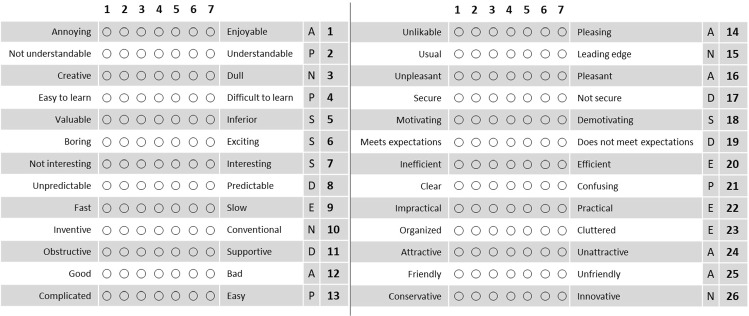
User Experience Questionnaire (UEQ) consisting of 26 questions for the six scales attractiveness (*A*), perspicuity (*P*), efficiency (*E*), dependability (*D*), stimulation (*S*), and novelty (*N*)

**Table:** 

Attractiveness	What is the overall impression?
Perspicuity	Is it easy to get familiar with and is it easy to learn?
Efficiency	Can the tasks be solved quickly and without unnecessary effort?
Dependability	Is the system reliable and does the user feel in control of its handling?
Stimulation	Is it exciting and motivating?
Novelty	Is the product innovative and catchy?

All points awarded by the participants were rescaled for the evaluation so that the possible range of points lies between $-3$ (extremely bad) and $+3$ (extremely good). A neutral evaluation usually lies between $-0.8$ and 0.8, whereas values $>0.8$ signify a positive evaluation and values $<-0.8$ a negative one [21]. Figure 4 shows all 26 UEQ questions for the six scales.

## Results

### Previous experience

Among the 21 participants, 12 reported having completed their third year and 9 their fourth year of medical studies. No participant reported having any previous experience with ECMO cannulation, though 9 participants had previous experience with other cannulation procedures (e.g., venous cannulation or venous catheter into hand/arm). There was no significant difference in training time or error count for either procedure when comparing the group with cannulation experience to the group without.

Five participants claimed to have used AR glasses once before; 3 of them reported having used a HoloLens 1. There was no significant difference between the group with previous AR experience and the group without any AR experience in terms of P1 training time or error counts in either P1 or P2. The training time of P2, however, was significantly lower for participants with previous AR experience ($p<0.01$, *t*-test). Of these 5 experienced participants, 2 performed P2 with AR instructions and 3 with conventional instructions.

### Overall performance

**Fig. 5:**
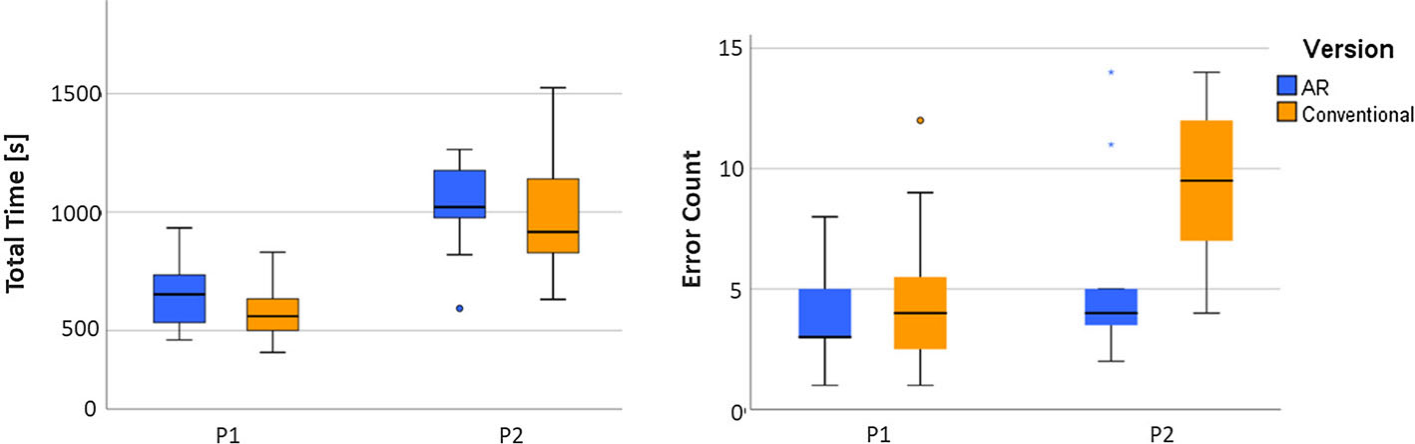
Training times and error counts for procedure 1 (P1) and 2 (P2)

Figure 5 shows the training times and total error counts for procedure 1 and procedure 2 for both AR-based and conventional instructions. Training times of both procedures are subject to a normal distribution (Shapiro–Wilk test), which is not the case for the error count. For P1, AR instructions were associated with slightly higher training times and slightly lower error counts. P2 was characterized by significantly higher mean training times than P1. For P2, AR instructions were associated with slightly higher training times, but with only half the variance. AR instructions also resulted in significantly lower error counts than conventional instructions ($p<0.05$, Mann–Whitney *U* test). Error counts and training times were not significantly correlated (Spearman correlation).

### Detailed error analysis

**Fig. 6:**
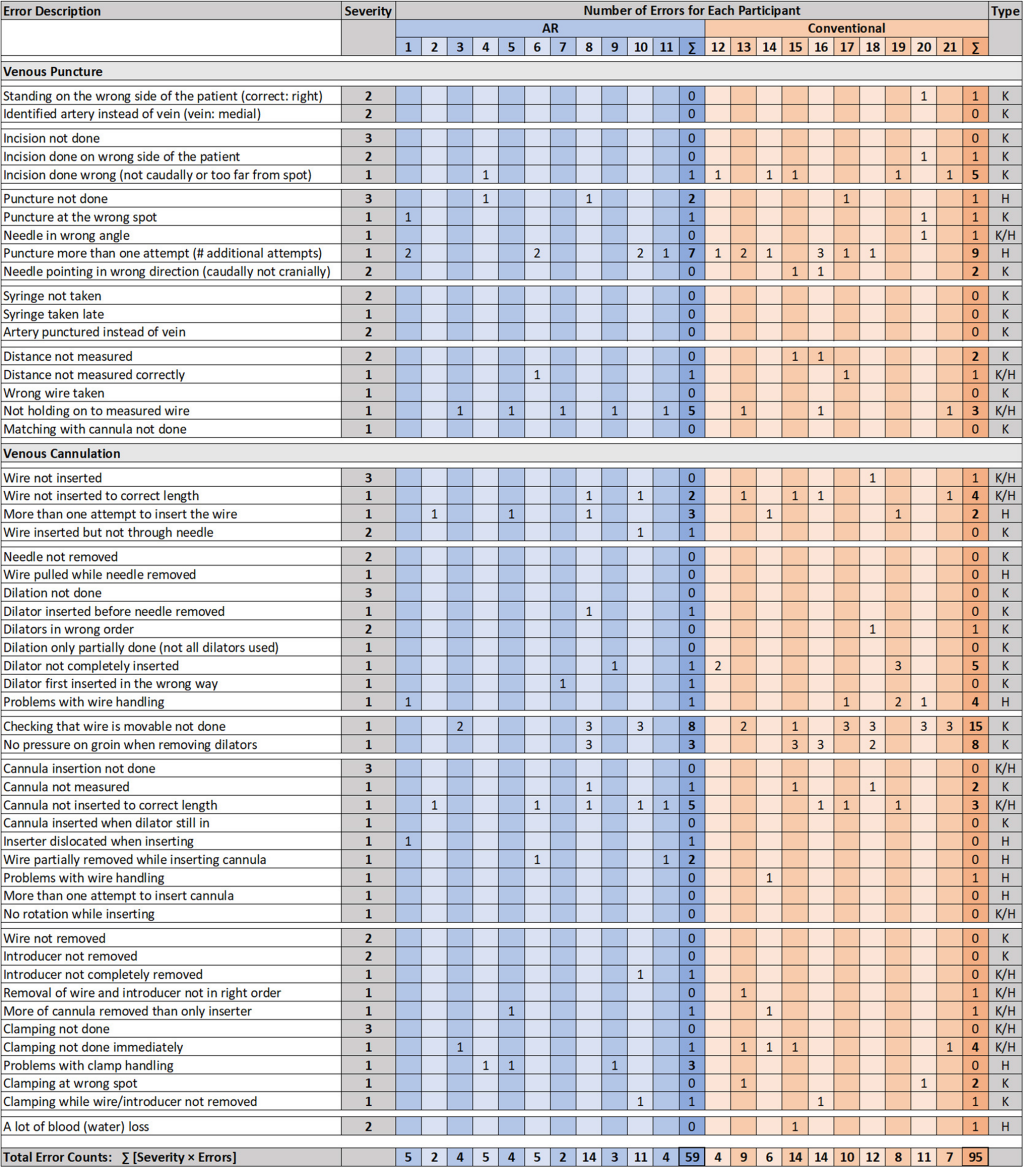
Full error protocol for P2 showing errors of each participant during AR-supported training (blue, $n = 11$) and conventional training (brown, $n = 10$). The rightmost column for each training type shows the error total. Total error counts are calculated by multiplying the errors in each row with the error severity factor, ranging from 1 to 3. The last column shows the error categorization into handling errors (*H*), knowledge errors (*K*), or a combination of both (*K*/*H*)

Figure 6 shows the full error protocol for P2. For the following comparisons, we rescaled errors associated with AR instructions to account for differences in participant numbers. Differences in errors occurred only for 1-point and 2-point errors. AR instructions resulted in 37% less 1-point errors, with 46 errors compared to 73 for conventional training. For 2-point errors, only one error was performed with AR, compared to 8 errors with conventional instructions. Both instruction types resulted in two 3-point errors.

Finally, we split the error counts according to their respective categories. Error counts for handling errors were similar for AR and conventional instructions, with a total of 21 each. The use of AR instructions resulted in a 66% decrease in error counts related to a lack of knowledge, reducing the error counts from 53 to 18. For those errors that could be related to both handling and knowledge, AR resulted in an error count of 15, compared to 21 for conventional instructions.

### User experience

**Fig. 7:**
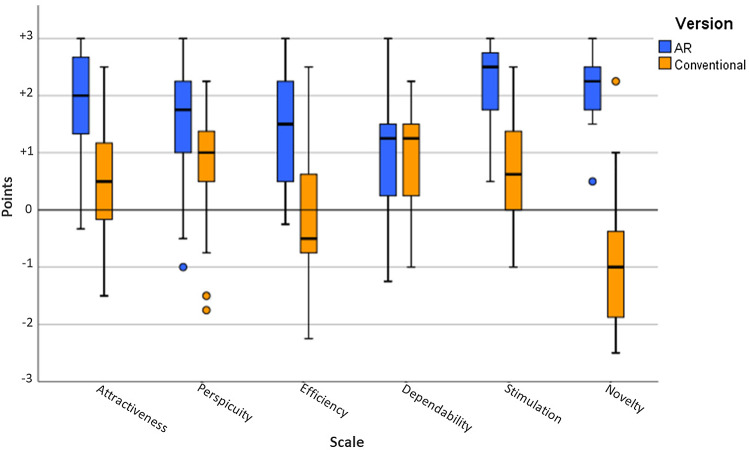
Point means per UEQ scale

The point ratings for the six scales of the UEQ questionnaire are visualized in Fig. 7. It is evident that the AR version was evaluated positively on all six scales (points >0.8). The conventional version has a positive evaluation for Perspicuity and Dependability, a neutral one for Attractiveness, Efficiency, and Stimulation ($-0.8>$ points $<0.8$), and a negative one for Novelty (points $< -0.8$). The best results for the AR version were obtained in the categories Attractiveness, Stimulation, and Novelty. The AR version performed considerably better in five of the six categories and only shows similar results when it comes to the category Dependability.

Differences in scores between the AR and the conventional version are significant for the categories Attractiveness, Perspicuity, Efficiency, Stimulation, and Novelty ($p<0.05$, Mann–Whitney *U* test). Clearly, not significant is the difference in Dependability.

## Discussion

Previous experience in cannulation was not shown to have any significant effect on participants’ performance. Although there was a significant difference in training times in P2 for participants with prior experience in AR, these participants were equally distributed between the group performing P2 with AR instructions and the one using conventional instructions. Therefore, we assume the influence of previous experiences to be negligible.

For the first procedure, no large differences in training times or error counts were found, suggesting that complexity was too low for differences in information presentation to have an impact on performance. Participants stated that they remembered relevant information from the initial video watched prior to the experiment and therefore often did not need to consult the instructions. For the more complex second procedure, instructions were consulted more frequently than in the first procedure but with important differences depending on the instruction type. Subjects using AR appeared to switch regularly between the displayed information and the point of execution, often evidenced by a brief pause in hand movement and sometimes by a brief but noticeable shift of the head. In contrast, subjects who used conventional instructions consulted the instructions less frequently but spent more time during each consultation. Differences in errors were mostly associated with knowledge errors, i.e., information that was missed during execution, which agrees well with our initial assumption about the benefits of having access to contextual information in close proximity.

Although we expected shorter training times for the procedures, this result is consistent with a previous study [3], which has attributed the differences to more engaging work with HMD. Similar to [16], we observed that videos were watched more frequently with the HoloLens than on a computer. The current state of the application may have also had an effect on training times. As videos were rather short, at less than 15 seconds, we did not implement a functionality for jumping to a specific point in the video. To watch a part of the video in AR again, participants had to replay it from the beginning, which was not the case for videos watched on the computer. Rather than watching the whole video in the AR instructions several times, participants only consulted long videos once or twice. As a result, error counts of the AR instructions were the highest for steps with the longest video sequences.

Deriving a detailed error protocol with error ranking and categorization has been shown to provide valuable insights on the effectiveness of training instructions. Error counts indicate that the significantly higher rankings in the UEQ were not only related to the novelty and excitement of using an OST-HMD, but were also linked to the much more convenient presentation of information. The UEQ suggests that participants found AR instructions motivating and exciting to use (Stimulation)—both desirable characteristics for frequent training and long-term retention—and rated them highly in terms of clarity of information and ease of learning (Perspicuity) and information acquisition speed (Efficiency). Dependability was expected to be slightly higher for the conventional instructions, since the likelihood of encountering technical difficulties was higher for the novel AR technology. Both instructions were rated positively with a score of over 1, even if voice commands or hand gestures sometimes posed difficulties.

While the results in this paper indicate a high potential of AR instructions for surgical training, they are based on a study with only 21 medical students. Further experiments would strengthen the validity of these findings. As described earlier, participants could only replay the full video sequences when using the AR instructions, and could not skip to the middle of a video when needed. Integrating this feature should further enhance the performance of AR instructions. To improve the realism of the training, currently missing steps like ultrasound verification or guidance should be integrated. Most importantly, this study only investigated the outcome of one training iteration and long-term training effects and the amount of information retained for future ECMO cannulations was not explored. A second iteration without any instructions could be of interest for future research, to investigate whether higher training times or lower error counts correlate with better performance in the long run. However, with respect to the high differences in error counts in P2, we don’t expect major changes in participants performances during a following assessment run, without feedback on previous performance. Future studies could therefore either perform a second training iteration with same instruction material and focus on how well performance improves during autodidactic training iterations. Alternatively, it could include a supervisor who provides feedback on participants’ performance, and then relate performance to the time spent with the supervisor. We expect participants to require significantly less supervision before achieving error-free execution when using AR for training.

## Conclusion

So far, there has been little research on how step-by-step instructions visualized by OST-HMD can be utilized to improve surgical training. In this paper, we demonstrated the potential of AR by taking the example of an ECMO cannulation. AR significantly reduced errors in the more complex second procedure and was clearly favored by the participants. Moreover, when comparing the variances in completion times and errors, AR instructions resulted in much more homogeneous performance levels. This is promising, as it helps to standardize training performance and makes training outcomes more predictable. We believe that these advantages of OST-HMD are generalizable to other surgical procedures. Further studies are needed to assess how AR training affects physicians’ long-term knowledge and skill development. For this purpose, it would also be interesting to utilize the integrated eye-tracking capabilities of recent OST-HMD, which allow for a more fine-grained analysis of participants’ behavior and their ongoing cognitive processes.
